# Civic engagement among orphans and non-orphans in five low- and middle-income countries

**DOI:** 10.1186/s12992-016-0202-8

**Published:** 2016-10-11

**Authors:** Christine L. Gray, Brian W. Pence, Lynne C. Messer, Jan Ostermann, Rachel A. Whetten, Nathan M. Thielman, Karen O’Donnell, Kathryn Whetten

**Affiliations:** 1 Department of Epidemiology, Gillings School of Global Public Health, CB #7435, University of North Carolina at Chapel Hill, Chapel Hill, NC 27599-7435 USA; 2 Center for Health Policy, Duke Global Health Institute, Box 90519, Duke University, Durham, NC 27708 USA; 3 School of Community Health, College of Urban and Public Affairs, Portland State University, PO Box 751, Portland, OR 97207 USA; 4 Department of Medicine, DUMC #3152, Division of Infectious Diseases and International Health, Duke University, Durham, NC 27710 USA; 5 Center for Child and Family Health, Duke University, 411 West Chapel Hill Street, Suite 908, Durham, NC USA; 6 Terry Sanford Institute of Public Policy, Box 90239, Duke University, Box 90239, Durham, NC 27708-0239 USA

**Keywords:** Orphans, Civic engagement, Low- and middle-income countries, Volunteer, Vote

## Abstract

**Background:**

Communities and nations seeking to foster social responsibility in their youth are interested in understanding factors that predict and promote youth involvement in public activities. Orphans and separated children (OSC) are a vulnerable population whose numbers are increasing, particularly in resource-poor settings. Understanding whether and how OSC are engaged in civic activities is important for community and world leaders who need to provide care for OSC and ensure their involvement in sustainable development.

**Methods:**

The Positive Outcomes for Orphans study (POFO) is a multi-country, longitudinal cohort study of OSC randomly sampled from institution-based care and from family-based care, and of non-OSC sampled from the same study regions. Participants represent six sites in five low-and middle-income countries. We examined civic engagement activities and government trust among subjects > =16 years old at 90-month follow-up (approximately 7.5 years after baseline). We calculated prevalences and estimated the association between key demographic variables and prevalence of regular volunteer work using multivariable Poisson regression, with sampling weights to accounting for the complex sampling design.

**Results:**

Among the 1,281 POFO participants > =16 who were assessed at 90-month follow-up, 45 % participated in regular community service or volunteer work; two-thirds of those volunteers did so on a strictly voluntary basis. While government trust was fairly high, at approximately 70 % for each level of government, participation in voting was only 15 % among those who were > =18 years old. We did not observe significant associations between demographic characteristics and regular volunteer work, with the exception of large variation by study site.

**Conclusion:**

As the world’s leaders grapple with the many competing demands of global health, economic security, and governmental stability, the participation of today’s youth in community and governance is essential for sustainability. This study provides a first step in understanding the degree to which OSC from different care settings across multiple low- and middle-income countries are engaged in their communities.

## Background

As adolescents emerge into young adulthood, their engagement in civic activities becomes an important foundation for their continued growth as well as their contributions to society throughout adulthood [1]. Communities and nations seeking to foster social responsibility in their youth are interested in understanding factors that predict and promote youth involvement in public activities [2, 3].

For many low- and middle-income countries (LMICs), the backdrop of the HIV/AIDS epidemic has not only increased care needs among citizens, but has compromised sustainable development through loss of its citizens who are in their productive years [4–6]. LMICs are also disproportionately affected by increasing numbers of orphans, in part due to the HIV/AIDS epidemic, and encouraging societal engagement of their youth is a critical aspect of their growth [7]. One of the concerns surrounding the AIDS epidemic in South Africa was the consequences for civil society of the growing number of orphans [8].

Civic engagement is increasingly recognized as important not only for democracy, but for health. A recent review argued that enhancing civic engagement would be required to achieve the global health components of the United Nation’s Sustainable Development Goals (SDGs), soon to replace the Millennium Development Goals (MDGs) [9].

While political engagement, such as voting, is often identified as the most tangible form of engagement, other activities such as volunteerism are also considered important. One definition specifies that “Civic engagement describes how an active citizen participates in the life of a community in order to improve conditions for a community’s future” [10]. In a study of civic education among 14 year-olds in 28 countries primarily in Europe, but including the U.S., China, Australia, Chile and Columbia, participant definitions of civic engagement included obeying the law, voting, participating in activities that benefit the community, promoting human rights, and following the news, among others, suggesting consistency across diverse contexts [11].

Few studies have described the prevalence and predictors of civic engagement in adolescents and young adults, particularly in LMICs. To our knowledge, none has examined civic engagement in orphans and separated children in LMICs. In many Western countries, particularly the U.S., exposure to any college-level education is a critical factor driving voter turn-out and volunteerism [12–15]. In the U.S., research on young people has indicated that being part of group settings increases knowledge and fosters social norms whereby participation is encouraged; such settings include faith-based institutions, schools, community organizations, and workplaces [16]. However, access to such opportunities is highly dependent on socioeconomic status (Flanagan [15]); parental education not only provides a mechanism for knowledge and awareness, but creates expected norms of engagement and greater ability to overcome costs associated with volunteering instead of working for pay [17]. In the Civic Education Study, schools were identified as particularly effective at fostering civic engagement when they had rigorous civic content, opportunity for speaking freely and openly, and a school culture that engendered participation [18]. Another analysis of the international Civic Education Study concluded that while home background strongly predicted civic knowledge and skills (largely through expected years of education and literacy), those differences were attenuated when predicting the likelihood of voting [11]. This study also examined subgroups for discrimination which may hinder civic engagement; attitudes toward rights for immigrants and women were fairly positive overall, though the study was unable to measure all the discrimination mechanisms which vary greatly by country [11]. In general, females demonstrated less knowledge but more positive attitudes toward immigrants and toward women’s rights than male counterparts [11].

OSC are a particularly unique population with respect to civic engagement because they are at particular risk of growing up without the aforementioned supports that promote access to civic awareness and activities. Studies of the impact of orphaning on schooling have shown both a significant negative impact in continuing education ([19, 20]) as well as recovery to participation in education equaling non-orphan counter parts after an expected period of lesser participation around the time of parental death [20, 21]. Importantly, a study of schooling in sub-Saharan Africa found that household resources, including the education of the head-of-household, critically influenced a child’s schooling, where as biological parents were much less critical [22]. However, the social safety net that confers those critical resources for OSC in many resource-poor countries is being stretched quite thin. Recent studies in China and sub-Saharan Africa have documented substantial needs for improvements in the safety net for orphan care because families do not have the resources to care for the growing number of orphans, the number of child-headed households is growing, and strong advocacy for deinstitutionalization is increasing pressure to eliminate one of the current options for alternative care [23–27].

It is unclear whether orphaning or separation leaves OSC vulnerable to disenfranchisement from society or whether their experience may facilitate a broader sense of community that promotes civic engagement, or whether that may vary by care setting or by characteristics such as being a single or double orphan. Based on existing literature, we hypothesize that prevalence of civic engagement will be higher among non-OSC than OSC and among single orphans than double orphans due to more limited resources among OSC and double-orphan OSC in particular. However, because prior literature has suggested that civic activities among youth are influenced by group settings that promote education and awareness of such activities, and because existing studies in this population and in other resource-poor settings have not observed differences in other outcomes related to health and mental wellbeing between OSC in institutional-based care and family-based care, we hypothesized that OSC in institution-based settings will have equal or higher prevalence of civic engagement indicators than OSC in family-based care [16, 28–30].

The Positive Outcomes for Orphans study (POFO) presents a unique opportunity to examine civic engagement among statistically representative samples of orphans in both institution-based care and in family-based care, as well as in a sample of non-orphans, in low- and middle-income countries. Here, we describe participation in activities such as volunteer service and voting, with the primary goal of estimating prevalence of these activities and exploring characteristics of adolescents who participate in them to provide an initial description of civic engagement among OSC in LMIC that may inform additional questions and research.

## Methods

### Study population

We used data from the Positive Outcomes for Orphans (POFO) study, a longitudinal cohort of institution-dwelling OSC, family-dwelling OSC, and non-OSC. For the parent POFO study, five countries were identified from a group of 13 countries in which the study team had prior relationships with community-based organizations interested in the research. Those five countries (including two distinct sites in India) were selected for their ethnic, political, geographic, cultural, and historic diversity. While these sites were not chosen specifically for their civic engagement differences, and while each is contextually distinct, all sites are in South or Southeast Asia or sub-Saharan Africa, and are struggling with a growing population of OSC under conditions of increasingly stretched safety net resources.

The six POFO study sites in five LMIC are: Battambang District, Cambodia; Addis Ababa, Ethiopia; Hyderabad, India; Nagaland, India; Bungoma District, Kenya; and Kilimanjaro Region, Tanzania. Children ages 6—12 were recruited at baseline between May, 2006 and February, 2008, depending on study site. OSC in institution-based care or family-based care were randomly sampled to be statistically representative of orphans in their respective setting at each of six study sites; non-orphans were sampled from the same geographic clusters to be a qualitative referent group. Additional details of the sampling frame are described elsewhere [31, 32].

At 90-month follow-up (approximately 7.5 years after baseline), participants who were at least 16 years old were asked questions about their civic engagement activities and trust in government. This analysis focuses on those older adolescents.

### Measures

#### Civic engagement

Given the dearth of studies on civic engagement in LMIC, we defined civic engagement to be consistent with existing work, especially since common themes emerged across many countries, even if those countries did not represent the resource-poor settings of interest in this study. Specifically, we defined civic engagement as the active involvement of community members in activities that contribute to community wellbeing, foster awareness of and participation in governance, or promote a sense of duty to help others. In the absence of existing work in this setting, we drew on existing measures from the Longitudinal Study of Adolescent Health in the United States so that our results had potential for comparability [33].

In participant interviews, we let OSC know we were interested in their unpaid participation volunteer and community work through organizations through service clubs, church and social action groups; we did not specify that we collectively termed these activities as ‘civic engagement’.

First, participants were asked whether they participated in regular community service or volunteer work between the ages of 12 to 18. They then were asked to specify whether it was strictly voluntary (because they wanted to do it) or whether it was required by caregivers, school, or a religious group. More than one answer to this question was accepted. Community and volunteer work is broadly defined to account for adolescents’ potentially limited opportunity (especially in varying cultural contexts) to participate in more traditional service activities or organizations; the emphasis is on unpaid efforts that serve to help or promote the wellbeing of others. Such activities may include helping a neighbor or community member outside the household with household work, volunteering at special events, participating in service days, providing instruction to other children, or other similar activities.

Additionally, participants were asked the degree to which they agreed with statements about trusting their local, state, and federal governments; the five-point scale ranged from “strongly agree” to “strongly disagree.” Though passive in nature, describing trust in government may be informative for understanding more active assessments of engagement and use of the existing measure enables potential for comparability to other settings.

For all sites, the voting age was 18. Respondents who were at least 18 were asked if they voted in the most recent government election. In addition, respondents 18 and older were asked whether they had ever tried to enlist in the military or militia. We coded this question as “Yes” if either or both were reported and “No” if neither was reported.

### Demographics

Gender, age, setting (institution-based care, family-based care, or biological family for non-orphans), and orphan status (single, double, or neither) were ascertained at baseline. Number of years of completed education was obtained at 90-month follow-up. Grade-for-age was used for modeling to better reflect the participant’s educational attainment and to reduce collinearity with age. For those 18 years or older, twelve years of education was assigned as the target grade-for-age; additional years beyond that were considered above target.

### Analyses

We calculated observed prevalences of the civic engagement variables described above. In addition, we constructed a predictive model of regular volunteer work (yes or no) using baseline demographics, current age, and current grade-for-age. Model parameters were estimated using Poisson regression with a log link. The model was weighted to incorporate the complex design of the sampling frame for orphans. Non-orphans (who were not sampled to be statistically representative) were assigned a weight of 1. Results are reported as prevalence ratios (PR) and 95 % confidence intervals (CI).

This study was approved by the Duke University Institutional Review Board (IRB) as well as IRBs at the individual study sites. Caregiver consent as well as child assent was obtained for all participants. Site-specific protocols were developed ensure mechanisms for child protection were in place in case of reports or observation of child maltreatment or neglect over the course of the study; interviewers were trained in these protocols.

All estimates were generated using Stata 13 [34].

## Results

The distribution of demographic characteristics among the 1,281 POFO study participants who were at least 16 years old at 90-month follow-up is shown in Table 1. There were 512 (40 %) institution-dwelling, 644 (50 %) family-dwelling OSC and 125 (10 %) non-OSC. Overall, 54 % were male and 60 % had no more than 9 years of education. For approximately 53 %, one parent was deceased for 25 %, both parents were deceased.

**Table 1:** Characteristics of older adolescents in the POFO population at 90-month follow-up

	Institution-based OSC		Family-based OSC		Non-OSC	
Characteristic	(*N* = 512)		(*N* = 644)		(*N* = 125)	
	*N*	%	*N*	%	*N*	%
Site						
Cambodia	42	8 %	92	14 %	12	10 %
Ethiopia	63	12 %	109	17 %	19	15 %
Hyderabad	132	26 %	129	20 %	29	23 %
Kenya	106	21 %	115	18 %	28	22 %
Nagaland	73	14 %	84	13 %	14	11 %
Tanzania	96	19 %	115	18 %	23	18 %
Gender						
Male	285	56 %	349	54 %	61	49 %
Female	227	44 %	295	46 %	64	51 %
OSC Status						
Both parents alive	84	17 %	76	12 %	125	100 %
Single orphan	225	44 %	453	70 %	0	0 %
Double orphan	203	40 %	115	18 %	0	0 %
Current age (years)						
16	120	23 %	160	25 %	50	40 %
17	164	32 %	197	31 %	48	38 %
18	131	26 %	181	28 %	22	18 %
> =19	97	19 %	106	16 %	5	4 %
Current education (years)						
< =7	115	22 %	229	36 %	30	24 %
8	71	14 %	69	11 %	11	9 %
9	114	22 %	122	19 %	34	27 %
10	89	17 %	99	15 %	21	17 %
11	62	12 %	56	9 %	15	12 %
> =12	48	9 %	48	7 %	9	7 %

About 45 % of subjects have participated in regular community service or volunteer work (Table 2). For those who have engaged in such work, 67 % did so out of their own volition and interest. They may also have participated for other reasons, including requirements by parents or caregivers (23 %), school (20 %) religious groups (7 %), or for other reasons (5 %). A very small minority (<1 %) did so due to a court order.

**Table 2:** Proportion of participants who participated in civic engagement activities

Question	Number	Percent
Regular participation in volunteer or community service		
No	652	55 %
Yes	525	45 %
If volunteered, was it required or strictly voluntary? (select all that apply)		
Strictly voluntary	350	67 %
Court ordered	3	1 %
Required by parents/caregiver	122	23 %
Required by school	106	20 %
Required by religious group	39	7 %
Other	27	5 %
Voted most recent election		
No	452	85 %
Yes	80	15 %
Enlist or serve: either military or militia		
No	1120	99 %
Yes	7	1 %
Trust: federal government		
Strongly Agree	301	26 %
Agree	485	42 %
Neither agree nor disagree	231	20 %
Disagree	63	6 %
Strongly Disagree	63	6 %
Trust: state government		
Strongly Agree	313	27 %
Agree	495	43 %
Neither agree nor disagree	217	19 %
Disagree	70	6 %
Strongly Disagree	54	5 %
Trust: local government		
Strongly Agree	315	27 %
Agree	495	43 %
Neither agree nor disagree	225	20 %
Disagree	74	6 %
Strongly Disagree	45	4 %

In terms of government trust, the distribution was similar for federal, state and local trust. About 70 % of participants either agreed or strongly agreed that they trust each level of government, and only 10–12 % (depending on government level) either disagreed or strongly disagreed that they trust the government.

Among the 532 participants who were at least 18 years old at 90-month follow-up and reported voting status, 80 (15 %) reported voting in the most recent election. For both 19 year-olds and 20-year olds, about 20 % and 23 %, respectively, voted. Among 18 year-olds, only 16 % reported voting. However, it is possible that those who were 18 at data collection were not 18 and eligible to vote in the last election.

The association of key demographic characteristics with report of regular volunteer work is shown in Table 3. Relative to Cambodia (the referent site), all sites had a lower prevalence of regular volunteer work. Nagaland and Hyderabad, the two sites in India, had particularly low prevalences of regular volunteer work compared to Cambodia.

**Table 3:** Predictive model of regular community service or volunteer work

Predictor	PR	95 % CI	p-value
Site			
Cambodia	1		
Ethiopia	0.48	(0.37,0.62)	0.000
Hyderabad	0.17	(0.12,0.25)	0.000
Kenya	0.63	(0.54,0.72)	0.000
Nagaland	0.06	(0.03,0.11)	0.000
Tanzania	0.72	(0.64,0.82)	0.000
Type			
Institution	1		
Family-based	1.08	(0.92,1.26)	0.376
Non-OSC	1.04	(0.82,1.32)	0.732
Gender			
Male	1		
Female	1.14	(0.97,1.33)	0.114
OSC Status			
Both parents alive	1		
Single orphan	1.06	(0.86,1.32)	0.560
Double orphan	1.07	(0.83,1.36)	0.613
Age (years)	1.05	(0.99,1.11)	0.123
Grade-for-age	1.01	(0.97,1.04)	0.719

We also found slight increases in the prevalence of regular volunteer work among females compared to males (PR = 1.14 [0.97,1.33]), family-dwelling OSC (PR = 1.08[0.92,1.26]) and non-OSC (1.04[0.82, 1.32]) relative to institution-dwelling OSC, single orphans (PR = 1.06[0.83, 1.36]) and double orphans (PR = 1.07[0.83, 1.36]) relative to having both parents alive, an additional year of age (PR = 1.05[0.99, 1.11]), and an additional year increase in grade-for-age (PR = 1.01[0.97, 1.04]); none of these were statistically significant, however.

## Discussion

We examined prevalence and predictors of service or volunteer work, voting, and trust in the government. We found that approximately 70 % of POFO participants reported trust in their local, state, and federal governments, though only 15 % of those eligible reported voting in the most recent election. However, nearly half (45 %) of adolescents reporting on service activities were engaged in regular volunteer work or community service. With the exception of site, we did not observe significant associations between key demographic factors and participation in regular community service or volunteer work, though the association with being female suggested a higher prevalence of regular service or volunteer work among females than males.

We observed substantial variation in in prevalence of regular service or volunteer work across sites, which may be due to differences in cultural expectations across countries or regions. The variation in estimates across sites may indicate that the relative importance of community service may be more or less emphasized in different cultures. This fairly crude measure may be better nuanced with different measures, or with improved understanding of what constitutes community contributions that would be analogous to more Western measures of civic engagement.

Though not statistically significant, females did report higher prevalence of community and volunteer work. This may indicate that the nature of available community service and volunteer work is more acceptably performed by females in some settings, or that the measure is failing to fully capture activities to which males in these settings may be contributing. It could also signal a need to better engage male adolescents and young adults in community-promoting activities.

Civic engagement is increasingly considered an important domain not only for sustainment of democracies, but for global health [9]. Participation in civic activities can increase social capital, and by extension, contribute to improved community health. For example, a recent study of the role of social capital in the performance of community health workers in Lao People’s Democratic Republic identified citizenship activities (such as engaging village elders or joining with others to solve a common problem) as particularly important for performance of community health workers [35].

The relationship between civic engagement and global health is also reciprocal: increased awareness of or participation in community health can foster broader civic engagement. For example, community and political engagement was increased after a network in Thailand of persons living with HIV/AIDS assisted in delivery of antiretroviral treatment to others in the community [36]. Similarly, civic engagement was increased in a group of Tanzanian youth randomized to an educational program that used health promotion of HIV/AIDS issues to foster self-efficacy and collective-efficacy around civic participation [37].

A key strength of this study is the novelty of both the question and the population. While some attention has been brought to the increasingly recognized importance of civic engagement, particularly among adolescents [3, 7], few studies have actually examined it. Furthermore, the role of orphans is especially relevant: their numbers are increasing [7], particularly in countries most affected by the global HIV/AIDS epidemic, where the need for community engagement and leadership is great. The POFO population is a statistically representative sample of both institution-dwelling and family-dwelling orphans, and includesa sample of non-orphans from six sites in five low- and middle-income countries. To our knowledge, this is the first study to examine these questions in orphans in LMICs. Finally, the longitudinal nature of the parent POFO study is a significant strength. While this analysis is limited to a single follow-up point due to the timing of introduction of relevant questions, the existing rapport developed by dedicated interviewers over time enabled responses from a hard-to-reach population.

Several weaknesses limit the results of this study. First, much of the POFO population has not yet reached the age of voting eligibility. Even those who were 18 at data collection may not have been eligible to vote in the most recent election prior to administration of the survey. Additional rounds of data collection will enable more robust results and greater capacity to examine associations with predictors as well as with longer-term health outcomes. Still, this initial examination is a useful first-step in understanding civic participation in this population. Additionally, the civic engagement questions were limited to the few broad-based questions presented here. We cannot tease out from these data whether, for example, participants vote because of obligation, desire for change, desire to support the government, or other reasons that may be helpful in understanding their motivations for engagement. Likewise, we do not know the reasons for non-participation. Though participants were asked about reasons for volunteering, the primary motivation is unknown. Furthermore, participants had varying opportunity to participate in civic activities depending on their age. While dependence on publically funded institutional support or subsidies could theoretically affect study participants’ willingness to express discontent with the government, the data on funding streams for support were unavailable, in part because funding sources for institutions and families were quite variable across source of organization providing funds/support (faith-based, non-profit, non-governmental organization, government), and across time (multiple different funding streams at different points in time). Only 4 institutions in the study were government run. We also do not have reason to believe the OSC had knowledge of the funding streams supporting them. Given the limited ability to pinpoint which type of funding was primarily driving resources at any given time and given that it is unlikely the OSC were aware of the funding streams supporting them, it is unlikely the participants were adjusting their responses based on such information.

Finally, the extant literature and instruments available to inform this study are limited primarily to European and U.S. contexts that may not be directly applicable to sub-Saharan Africa and South and Southeast Asia. Our study provides an important first look at describing participation in activities widely thought to be important regardless of country, but key indicators of civic engagement in these settings may be quite different.

## Conclusion

As the world’s leaders grapple with the many competing demands of global health, economic security, and governmental stability, the participation of today’s youth in community and governance is essential for sustainability. We hope this study motivates additional focus on civic engagement of adolescents to understand what promotes civic engagement in their communities, which may contribute to both sustainable development as well as global health.

## Abbreviations

AIDS: Acquired immune deficiency syndrome
CI: Confidence interval
HIV: Human immuno-deficiency virus
IRB: Institutional review board
LMIC: Low and middle-income countries
MDG: Millennium development goals
OSC: Orphans and separated children
POFO: Positive outcomes for orphans
PR: Prevalence ratio
SDG: Sustainable development goals
U.S: United States
